# P2CS: a two-component system resource for prokaryotic signal transduction research

**DOI:** 10.1186/1471-2164-10-315

**Published:** 2009-07-15

**Authors:** Mohamed Barakat, Philippe Ortet, Cécile Jourlin-Castelli, Mireille Ansaldi, Vincent Méjean, David E Whitworth

**Affiliations:** 1 CEA, DSV, IBEB, LEMiRE, CNRS, Université Aix-Marseille II, CEA Cadarache, F-13108 Saint-Paul-lez-Durance, France; 2 Laboratoire de Chimie Bactérienne, CNRS, F-13402 Marseille, France; 3 Institute of Biological, Environmental and Rural Sciences, Aberystwyth University, Ceredigion, SY23 3DD, UK

## Abstract

**Background:**

With the escalation of high throughput prokaryotic genome sequencing, there is an ever-increasing need for databases that characterise, catalogue and present data relating to particular gene sets and genomes/metagenomes. Two-component system (TCS) signal transduction pathways are the dominant mechanisms by which micro-organisms sense and respond to external as well as internal environmental changes. These systems respond to a wide range of stimuli by triggering diverse physiological adjustments, including alterations in gene expression, enzymatic reactions, or protein-protein interactions.

**Description:**

We present P2CS (Prokaryotic 2-Component Systems), an integrated and comprehensive database of TCS signal transduction proteins, which contains a compilation of the TCS genes within 755 completely sequenced prokaryotic genomes and 39 metagenomes. P2CS provides detailed annotation of each TCS gene including family classification, sequence features, functional domains, as well as genomic context visualization. To bypass the generic problem of gene underestimation during genome annotation, we also constituted and searched an ORFeome, which improves the recovery of TCS proteins compared to searches on the equivalent proteomes.

**Conclusion:**

P2CS has been developed for computational analysis of the modular TCSs of prokaryotic genomes and metagenomes. It provides a complete overview of information on TCSs, including predicted candidate proteins and probable proteins, which need further curation/validation. The database can be browsed and queried with a user-friendly web interface at .

## Background

His-Asp phosphorelays, or two-component system (TCS) signal transduction pathways, are found across all three domains of life, allowing adaptive responses to changes in environmental conditions. However, they are mainly found in bacteria where they control diverse aspects of bacterial metabolism, such as cell differentiation, morphogenesis, central metabolism, motility, biofilm formation and virulence. These systems were classically described as the association of two proteins that communicate through a His-Asp phosphorelay [1]. A typical TCS comprises a histidine kinase (HK) sensor protein, which is capable of autophosphorylation on a conserved His residue, before transferring the phosphoryl group to a conserved Asp residue within the receiver domain (REC) of a response regulator (RR). This 2-step phosphorelay constitutes the basic scheme of TCS signalling. More complex systems utilise a 4-step phosphorelay (His_1_-Asp_1_-His_2_-Asp_2_) that is made possible by the addition of 2 intermediate phosphorylation domains: a second receiver domain homologous to that of RRs, and a phospho-His domain, called HPT (for Histidine Phosphotransfer), which is the phosphodonor for the terminal RR protein of the pathway. In 4-step phosphorelays, the phosphoacceptor domains can be found distributed across 2 to 4 individual proteins [2].

The large number of TCS protein sequences available demands user-friendly databases to facilitate inter-genomic and intra-genomic analyses. Currently, databases describing prokaryotic TCSs contain data from only a subset of available genomes (eg. SENTRA, Genome Atlas) [3,4], or analyze TCSs on the basis of predicted proteins (eg. MiST) [5]. However, valuable data are often overlooked by protein prediction tools, and, to our knowledge, no database is currently available for performing analysis of metagenomic TCSs.

We have therefore developed a novel resource, the P2CS database, which contains the TCSs of all available bacterial and archaeal genomes, and 39 microbial metagenomes. Our objective was to provide an easy to use environment for exploitation by users, with the data being completely available and consultable by all of the scientific community.

## Construction and content

### Database description

**Figure 1:**
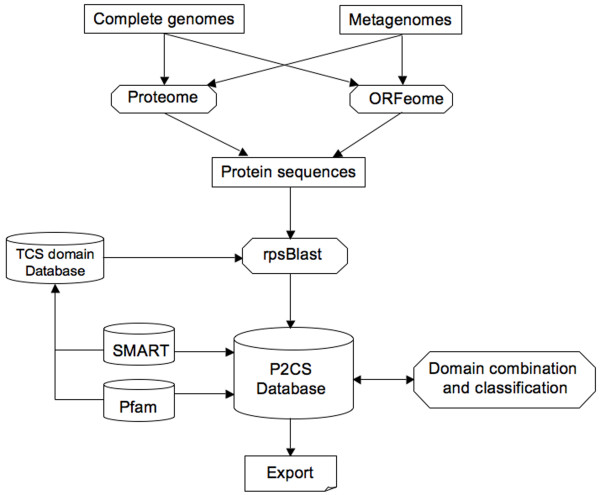
**Schematic modular pipeline of P2CS**. After data download of prokaryotic genomes and metagenomes, protein sequence files are constructed and searches for TCS domains performed. Finally each TCS protein is annotated and categorized into a TCS family.

The modular architecture of P2CS is described in Figure 1. Prokaryotic TCSs were defined through a comprehensive computational analysis of 755 completely sequenced genomes and 39 metagenomes. Genome data were downloaded from the NCBI [6] and metagenomic data were downloaded from the IMG/M [7]. The P2CS pipeline is designed to take protein and whole replicon genomic files as input. In the second case the DNA sequence is scanned to predict the entire set of valid ORFs, defined as the DNA segments occurring between two stop codons and exceeding 100 nucleotides, with no further assumption about the presence or not of coding sequences. Thereafter the sequences are translated to constitute the ORFeome. This approach allows identification of possibly mis-predicted (overlooked) TCS proteins, by comparison to the predicted TCS from the proteome pool.

The identification of TCS candidates was accomplished by domain analysis of each predicted protein. The pool of domains used to search for TCS proteins was manually selected from the literature [4,5,8-11] and extracted from within the Pfam and SMART libraries. All the data are stored in the P2CS database, accessible via our web interface, GenoBrowser. The P2CS pipeline was developed to search the numerous combinations of TCS modules and to categorize TCS proteins into families based on similarity and/or domain architecture. Our process identifies a subset of TCSs as 'probable incomplete TCS proteins'. This is the case when a HATPase (HK ATPase) domain is identified in a protein, without the typical N-terminal His-containing phosphoacceptor site (HisKA domain). These incomplete HKs (IHKs) were then analysed for the presence of a probable site of phosphorylation (H-box). First, for all the predicted HKs belonging to each replicon, we calculated the maximal and minimal distances separating the N-terminal and the C-terminal extremities of, respectively, identified HisKA domains and HATPase domains. This interval was then used to search for putative phospho-accepting His-residues in the IHKs. Secondly, searches for the site of phosphorylation were undertaken by constructing alignments of the 10 amino acid residues surrounding the conserved histidine from all predicted HKs in each replicon, and using the alignment to create a profile. We compared each predicted HK to the profile and calculated a score, which allowed the definition of a minimal value (min score). The same process was repeated for each IHK and in the presence of a putative H-box (below the min score), an IHK was reclassified as a HK. As in other studies [3,5,12], proteins belonging to the GyrB, MutL or HtpG family were excluded.

During the development of P2CS, we noted the presence of enzymes erroneously classified as TCS proteins and containing domains involved in TCS signal transduction. To circumvent this problem, we manually compiled a list of enzyme activities (Table 1) that we determined as being potentially present in TCS proteins, from the ENZYME database [13] and compared our dataset to this list. The list included enzyme activities known to be involved in TCS signal transduction (eg. histidine kinase activity) and enzymes found as accessory domains in true TCS proteins (eg. sulfate-transporting ATPase or STAS domain).

**Table 1:** Enzyme list implicated in TCS signal transduction

EC Number	Enzyme Description	Present In TCS
2.7.13.3	Histidine kinase	Yes

3.1.1.61	Protein-glutamate methylesterase	Yes

2.1.1.80	Protein-glutamate O-methyltransferase	Yes

4.6.1.1	Adenylate cyclase	Yes

4.6.1.2	Guanylate cyclase	Yes

3.1.3.3	Phosphoserine phosphatase	Probable

3.6.3.25	Sulfate-transporting ATPase	Probable

2.7.11.25	Mitogen-activated protein kinase kinase kinase	Probable

2.7.11.17	Calcium/calmodulin-dependent protein kinase	Probable

3.1.3.16	Phosphoprotein phosphatase	Probable

3.4.21.53	Endopeptidase La	Probable

1.8.1.9	Thioredoxin-disulfide reductase	Probable

3.6.3.28	Phosphonate-transporting ATPase	Probable

4.1.1.18	Lysine decarboxylase.	Probable

1.4.1.3	Glutamate dehydrogenase (NAD(P)(+))	Probable

4.1.1.19	Arginine decarboxylase	Probable

4.1.1.17	Ornithine decarboxylase	Probable

All the identified enzymes that did not match with the selected enzyme list were discarded. For instance, in *Burkholderia phymatum STM815*, the protein Bphy_5251 contains a REC domain and is annotated as a response regulator in GenBank. This protein seems, however, to be more probably involved in an ABC-type transport system.

Finally, the cellular localization of each TCS protein was determined by the presence or absence of transmembrane (TM) segments, using the HMMTOP predictor [14].

**Figure 2:**
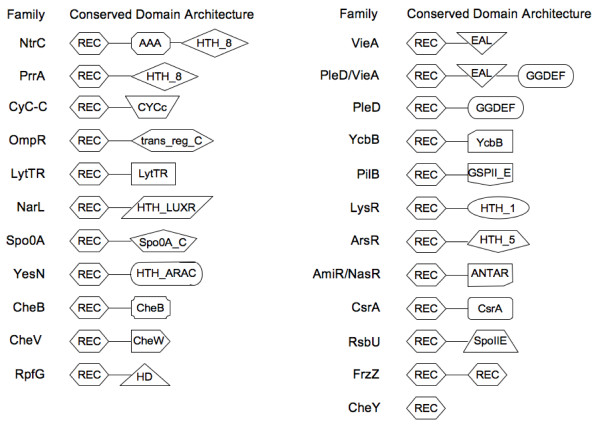
**Response regulators classification**. Schematic representation of the conserved domain architectures of RRs.

**Figure 3:**
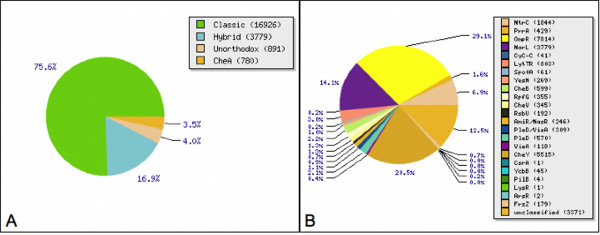
**Numbers and types of TCS proteins**. Histidine kinases (A) and response regulators (B), in prokaryotic genomes and metagenomes.

**Figure 4:**
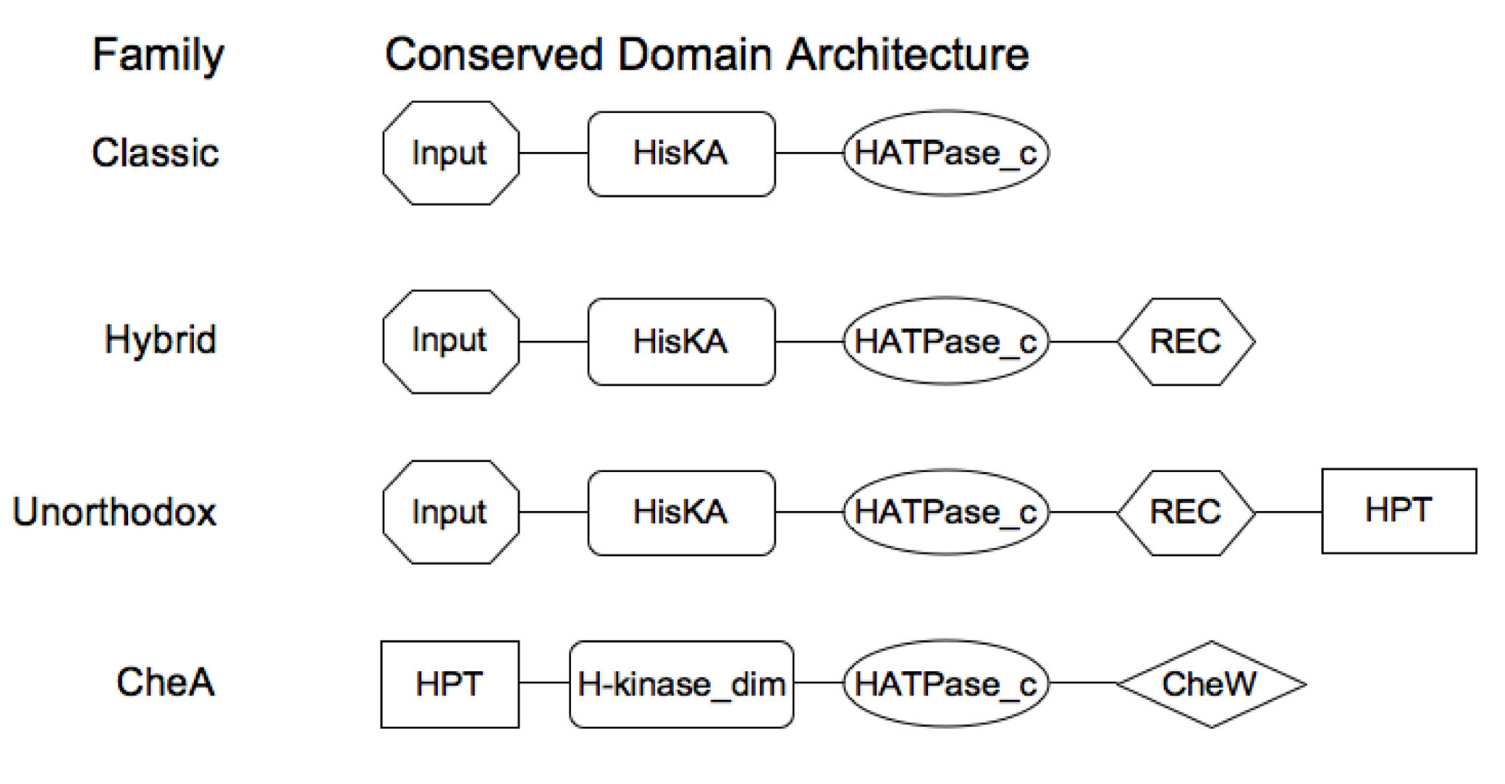
**Histidine kinases classification**. Schematic representation of the conserved domain architectures of HKs.

TCS proteins can be classified according to their domain architecture [10,15]. RR proteins are divided into distinct families on the basis of the possession of specified accessory domains. The majority of RRs comprises an N-terminal receiver domain and a C-terminal signal output domain, although more complicated combinations exist. The P2CS process classified RRs in 24 different families (Figure 2). The most abundant families, across all genomes, are OmpR, CheY and NarL (Figure 3b). A previous study on 200 prokaryotic genomes [10] showed similar results and an exploration of the KEGG database [16] shows that the OmpR and NarL families are particularly large and well-studied. The most common domain architecture of HKs consists of single/multiple input domains, with a single transmitter domain. The P2CS classification of HKs considers the presence/absence of receiver, HPT and/or CheW domains. HKs are categorized as classic, hybrid, unorthodox or CheA family (Figure 4), while proteins with HPT or HisKA domains, but no HATPase or receiver domains, are classified as phosphotransfer proteins.

The current version of P2CS contains 53233 TCS proteins, which comprises 22376 HKs (Figure 3a), 26892 RRs (Figure 3b) and 1900 phosphotransfer proteins. The ORFeome search allows retrieval of a further 774 supplemental TCS proteins from completely sequenced genomes.

### Data validation

To test the performance of P2CS, a variety of approaches were taken. P2CS data were compared to different analyses available in the literature generated using other search methods. The accuracy of P2CS in identifying TCS proteins was compared to several studies using manual curation to detect TCSs [11,17-20]. We used these studies as reference sets and calculated sensitivity, specificity and precision to assess the efficiency of the P2CS analysis (Table 2). The results show that automated P2CS retrieval, gave very similar results to manual curation (sensitivity, specificity and precision means of 99.1, 99.9 and 97% respectively). In some cases more TCS proteins were identified by P2CS compared to manual analysis, with the extra proteins primarily being identified through ORFeome searches.

**Table 2:** Performance test of P2CS

Species	Manually defined TCS proteins	P2CS Predicted TCS proteins	Sensitivity (%)	Specificity (%)	Precision (%)	Reference
*Anaeromyxobacter dehalogenans 2CP-C*	174	188 (185+3)	100	99.67	92.55	[15]

*Bacillus anthracis str. Ames*	102 (94+8)	104 (94+10)	100	99.96	97.9	[18]

*Bacillus anthracis str. Sterne*	102	103	99.02	99.96	98.06	[18]

*Bacillus cereus ATCC 14579*	101 (99+2)	102 (99+3)	100	99.98	99.02	[18]

*Bacillus cereus ATCC 10987*	101 (100+1)	99 (98+1)	98.02	100	100	[18]

*Bacillus cereus E33L*	107	107	100	100	100	[18]

*Bacillus subtilis*	70	70	100	100	100	[18]

*Bacillus thuringiensis serovar konkukian str. 97-27*	109	109	100	100	100	[18]

*Escherichia coli str. K-12 substr. MG1655*	62	62 (61+1)	100	100	100	[15]

*Myxococcus xanthus DK1622*	278 (276+2)	283 (281+2)	99.28	99.91	97.53	[15]

*Nitrosospira multiformis ATCC 25196 chromosome 1*	62	59	93.55	99.96	98.31	[19]

*Pseudomonas syringae pv. syringae B728a*	142	143	97.89	99.92	97.2	[11]

*Pseudomonas syringae pv. tomato str. DC3000*	143 (140+3)	144 (139+5)	97.2	99.92	96.53	[11]

*Pseudomonas syringae pv. phaseolicola 1448A*	139 (137+2)	141 (138+3)	97.84	99.87	96.45	[11]

*Sorangium cellulosum So ce56*	267	273	98.88	99.92	96.70	[15]

*Streptomyces coelicolor A3(2)*	164	187	99.39	99.68	87.17	[17]

*Xanthomonas campestris pv. campestris ATCC 33913*	106	110	100	99.9	96.36	[20]

*X. campestris pv. campestris 8004*	106	110	100	99.9	96.36	[20]

*X. axonopodis pv. citri 306*	114	120	100	99.86	95	[20]

*X. campestris pv. vesicatoria 85-10*	121	126	100	99.86	95	[20]

*X. oryzae pv. oryzae KACC10331*	92 (91+1)	96 (95+1)	100	99.9	95.83	[20]

*X. oryzae pv. oryzae MAFF 311018*	93	95	100	99.95	97.89	[20]

Comparison to manually detected TCS proteins (numbers in parentheses are the details of predicted and mis-predicted TCS proteins). Parameters calculation: Sensitivity = TP/TP+TN, Specificity = TN/TN+FP, Precision = TP/TP+FP.

TP. True positive, TN. True negative, FP. False positive, FN. False negative.

**Figure 5:**
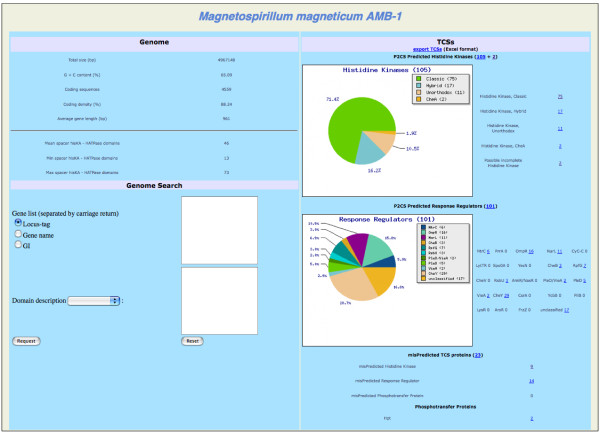
**P2CS analysis of the *Magnetospirillum magneticum AMB-1 *genome**. Each class result provides a link to a detailed gene list. Clickable links are underlined.

**Figure 6:**
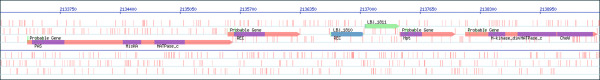
***Leptospira borgpetersenii serovar Hardjo-bovis JB197 *genome**. Genomic context for a chromosomal region (2133100 – 2139600). Genes are represented in the six reading frames with initial data from the NCBI database (green genes with domains in blue) and the result of the P2CS analysis process (genes in light red with domains in purple). Red vertical lines represent stop codons and green lines represent potential start codons.

To bypass the problem of gene underestimation during the gene prediction process, P2CS constitutes a searchable ORFeome (see above). This original aspect of the method allows the recovery of nearly 2% more TCS proteins compared to searches on the equivalent proteomes. An interesting example comes from *Magnetospirillum magneticum AMB-1*, with 23 mis-predicted TCS proteins identified in addition to the 208 TCS proteins defined after proteome scanning (Figure 5). Another spectacular example comes from *Leptospira borgpetersenii *with a total of 52 TCS proteins initially identified and a further 16 mis-predicted proteins detected using the ORFeome. Figure 6 shows an illustrative example, a genomic context of six TCS genes, of which four were retrieved by the ORFeome process. Indeed, a large proportion of the TCS genes P2CS identified as 'mis-predicted TCS genes' were found to lie adjacent to other TCS genes, suggesting that they encode true TCS proteins. All the mis-predicted proteins were manually checked.

To facilitate the visualization of metagenomes, the different contigs of each metagenome were joined and rebuilt into an artificial and linear chromosome. The new positions of genes were then recomputed to allow exploration of genomic contexts. The transition between two different contigs is announced to avoid misinterpretation of gene organisation.

IHKs in all genomes and metagenomes were not eliminated during the screening process and can therefore be curated by users. Additionally, mis-predicted TCS proteins can be visualized as the longest possible coding sequence, so that users can also make their own start assignments.

## Utility and discussion

P2CS provides an integrated environment for exploration, visualization and annotation of TCS proteins from all available bacterial genomes and metagenomes via [21]. The P2CS homepage contains a navigation bar that allows database browsing. Among the menus, users will also find P2CS Browse, which links directly to sortable lists of analysed genomes, plasmids and metagenomes. The selection of a microbe or a microbiome displays the result of the P2CS analysis process. It shows global counts of the different categories of TCSs and detailed class counts of each category. Each class result provides a clickable link to a detailed gene list. Selecting an object from the list of identifiers, displays a detailed gene description page with an image representing the gene in its genomic context, in the appropriate frame. Blast searches can be performed with the gene using external links, against the NCBI protein database or the annotated databases Swiss-Prot/TrEMBL. To obtain detailed information on a given gene, the software provides database links to investigate structural and functional domains of the corresponding protein sequence using the Conserved Domain Search service [22], the Simple Modular Architecture Research Tool [23] and the TMHMM transmembrane topology prediction method [24]. The presence and location of signal peptide cleavage sites in amino acid sequences can also be checked using SIG-Pred [25].

A second menu, P2CS Search, provides several search modes that allow users to request genes on the basis of their locus-tag, domain possession or TCS class. The search module builds search output as a tabular view that is linked to a full description and genomic context for each selected gene. The gene description page is the core exploration tool, providing several analysis options as described above. Analysis of each gene can be performed and users have the ability to display and propose the modification of any gene.

P2CS was designed to allow download of TCS data in tab-delimited format and generates a file compatible with spreadsheet programs such as Excel. For each genome and metagenome, users can also download the flat format files used for the construction of the database.

P2CS has been developed for computational analysis of the modular TCSs of prokaryotic genomes and metagenomes. It provides a complete overview of information on TCSs, including predicted candidate proteins and probable proteins, which need further curation/validation. The analysis process recovers each protein presenting N-terminal HisKA or C-terminal HATPase domains and classifies them as probable incomplete HK. The status can be changed through the manual curation process.

Users can modify annotation parameters and append comments, which are made available for consultation by other users. To ensure the integrity of the database, we propose to the interested experts to download formatted data and then after manual curation, the same downloaded files can be used as exchange format for an update of the database by the P2CS team.

One of the most important features of P2CS is the ability to search for TCSs within an ORFeome. One common problem of prokaryotic genome annotation is the accuracy of gene prediction and the loss of valuable data as a consequence of underestimation of the number of predicted genes. A blatant example is the genome of *M. magneticum AMB-1 *[26], with 23 overlooked TCS genes. A possible explanation for the high number of missing TCS genes is the GC richness of this genome (65%), which may constitute a complication in the gene prediction process.

## Conclusion

Biologists with little or no computing background, have an increasing need for fast and intuitively usable tools, which is why P2CS has been developed as an interactive system for editing and viewing TCS information.

The current P2CS database contains information on over 53000 predicted TCS proteins. The pipeline used to predict TCSs begins with a domain annotation of all proteins from completely sequenced genomes and metagenomes, searches the numerous combinations of TCS modules and classifies TCS proteins. The P2CS database analyses TCSs in both predicted proteomes and reconstituted ORFeomes. This last process is a unique feature of our system, which allows the recovery of nearly 2% more TCS proteins.

Databases devoted to TCSs are major resources for the signal transduction research community but these currently do not include metagenomic data. P2CS is the first database of its kind that provides metagenomic TCS information, with nearly 17% of identified TCS proteins originating from metagenomes.

P2CS is an open resource for biologists and results are presented for user exploration as an interactive web interface. Currently, our database contains a high quality automatic analysis of the TCSs of all currently available genomes and metagenomes, including 8 bacterial genomes (*Escherichia coli, Bacillus subtilis*, *Shewanella oneidensis *and 5 myxobacterial strains) curated manually by our team.

## Availability and requirements

P2CS is publicly available at [21]. It runs on most web browsers, including Mozilla Firefox, Safari and Internet Explorer.

## Authors' contributions

MB and PO developed and designed the database. CJ, MA and DW participated in the improvement of the database functionalities. CJ, MA, VM and DW validated model strains data. MB drafted the manuscript. PO, MA and DW revised the manuscript. All authors have read and approved the final submitted version of this manuscript.
